# Barriers and facilitators to dental care access among asylum seekers and refugees in highly developed countries: a systematic review

**DOI:** 10.1186/s12903-020-01321-1

**Published:** 2020-11-25

**Authors:** Martha Paisi, Rebecca Baines, Lorna Burns, Anastasios Plessas, Philip Radford, Jill Shawe, Robert Witton

**Affiliations:** 1 Peninsula Dental Social Enterprise (Derriford Dental Education Facility), University of Plymouth, 20 Research Way, Plymouth, PL6 8BT UK; 2 School of Nursing and Midwifery, University of Plymouth, Drake Circus, Plymouth, PL4 8AA UK; 3 Centre for Health Technology, University of Plymouth, Drake Circus, Plymouth, PL4 8AA UK; 4 Peninsula Dental School, University of Plymouth, Drake Circus, Plymouth, PL4 8AA UK; 5 Rotherham NHS Foundation Trust Community Dental Service, New Street Health Centre, Upper New Street, Barnsley, S70 1LP UK

**Keywords:** Asylum seeker, Refugee, Displaced persons, Access, Dental care

## Abstract

**Background:**

Dental diseases are prevalent among asylum seekers and refugees (ASRs). Despite significant treatment needs, access to dental care in host countries is often limited. The aim of this systematic review was to identify the barriers and enablers to dental care access for ASRs in host countries of very high development.

**Methods:**

Five health and social care databases and eight grey literature sources of information were searched. The Critical Appraisal Skills Programme tool was used to critically appraise included studies. Thematic analysis was undertaken to identify common themes. These were then deductively organised according to Penchansky and Thomas’s modified access model. All review stages were conducted by two independent reviewers.

**Results:**

Nine papers were included in the review. ASRs encounter significant challenges to accessing dental care in their host countries. These include affordability, communication difficulties, insufficient interpretation, limited knowledge of the healthcare systems and healthcare rights, and negative encounters with healthcare teams. The views and experiences of dental care teams providing care to ASRs were explored in only one study.

**Conclusions:**

Both population and healthcare characteristics influence access to dental care for ASRs. Affordability, awareness and accommodation are most frequently described as barriers to dental access for this population. The diverse needs of this population need to be recognised by policy makers, commissioners and practitioners alike. Cultural competence needs to be incorporated into dental services and any interventions to improve access to dental care for this population.

Registration

PROSPERO- International prospective register of systematic reviews (CRD42019145570).

## Background

The United Nations Refugee Agency (UNHCR) is witnessing the highest level of forced displacement on record, with 70.8 million people displaced worldwide due to war, persecution, violence, and violations of human rights [1]. Among the people of concern for UNHCR, are asylum seekers and refugees (ASRs). As per the UNHCR’s 1951 Geneva Convention, a refugee is defined as a person who ‘*owing to a well-founded fear of being persecuted for reasons of race, religion, nationality, membership of a particular social group, or political opinion, is outside the country of his nationality, and is unable to or, owing to such fear, is unwilling to avail himself of the protection of that country’* [2]. An asylum-seeker (with ‘pending case’) is defined as an ‘*individual who has sought international protection and whose claim for refugee status has not yet been determined’* [3]. At the end of 2018, there were 25.9 million refugees (20.4 million refugees under UNHCR’s mandate) and 3.5 million asylum seekers, with developed countries hosting 16% of the global refugee population [1]. Developing regions shoulder a disproportionately larger responsibility (84%), with one third of the global refugee population (approximately 6.7. million people) being hosted in the least developed countries [1].

The majority of the global refugee population are hosted in poorly resourced countries. However, inequalities in health outcomes and healthcare among this group across a number of services also exist in high-income countries [4–6]. This raises the importance of addressing this group’s health needs in highly-developed countries. Oral diseases, particularly dental caries and periodontal disease, are highly prevalent among ASRs [7]. Disease levels in this population are consistently higher, even when compared to the most vulnerable population groups in the host countries [7, 8]. For example, a study that analysed existing data in Australia, found that refugees had a mean number of untreated decayed teeth between 2.0 and 5.2, whilst for the general population this ranged from 0.6 to 1.4.[8]. Refugees also had fewer restored teeth (1.0–5.8 as compared to 4.1–9.3 for the general population). In the same study, disproportionate differences were found when the number of decayed, missing and filled teeth of refugees was compared to that of ‘special needs groups’, such as recipients of public benefits, in Australia [8]. Another study that compared oral health between refugee and US children, showed that refugee children, primarily those from Eastern Europe, were more likely to have untreated caries than white US children (OR: 9.4, 95% CI 6.06–14.7) [9].

Universal access to quality healthcare is an important indicator of a healthcare system’s performance [10]. However, as suggested by Keboa et al. [7], despite significant treatment needs, ASRs’ access to, and utlisation of dental care in their host countries is severely limited. A review of dental services for refugees in Australia showed that this group’s pattern of service use did not reflect their needs [11].

Access is a complex notion as indicated by the variety of interpretations attached to the concept [12, 13]. It is commonly defined as the interplay of factors influencing entry into or use of a healthcare system [14]. While some authors conceptualise access with an emphasis on the characteristics of healthcare services influencing utilisation of care [15], others view it within the ability of populations to seek and obtain care [16]. There are also those who regard access as an interplay between population and health system characteristics [14]. In our review, access is conceptualised within the Penchansky and Thomas’s model [14] where access is described as the degree of ‘fit between a patient and the healthcare system’ influenced by specific dimensions including ‘accessibility, availability, acceptability, affordability, and accommodation’ [14].

Considering the acknowledged burden of oral diseases among ASRs and the reported disparity between service need and use [11], identifying barriers and facilitators to this group’s access to dental care is of paramount importance to ensure that services maximise their potential benefits and effectively respond to the needs of this particular group [17]. A scoping review by Keboa et al. [7] into the oral health of ASRs identified the ‘healthcare system, society, and personal oral health beliefs and behaviours’ as the main factors influencing access to and utilisation of dental services for this population. However, this review provided only a brief description of access to and utilisation of services by this population. To our knowledge, no systematic review investigating the barriers and facilitators to dental care access among ASRs in highly developed countries has been conducted, highlighting the unique contribution of this work.

This systematic review aimed to address the question: What are the barriers and facilitators to dental care access among ASRs in highly developed countries?

## Methods

### Protocol and registration

The review protocol is registered on the International prospective register of systematic reviews- PROSPERO (CRD42019145570).

### Eligibility criteria

Eligibility criteria used at the title/abstract and full text-level, included the following (Table 1):

**Table 1:** Eligibility criteria

Population/participants	People seeking asylum, refugees and those under subsidiary or humanitarian protection (as per the UNHCR’s 1951 Geneva convention definition), dental healthcare professionals and other stakeholders working with, or supporting this population group
Phenomenon of interest	Experience of displacement
Outcomes	Barriers and facilitators to accessing dental care
Types of study	Inclusion: Qualitative research studies (including those which are components of mixed methods evaluations if the qualitative findings were clearly distinguishable) Exclusion: Quantitative studies (without qualitative component), narrative reviews, letters, commentaries and editorials, conference proceedings
Location of study	Inclusion: Studies conducted and focusing on access to dental care in countries of very high Human Development Index (HDI) [18] (Additional file 1) Exclusion: Studies conducted in countries of high, low or medium HDI

We included only qualitative research studies as they utilise the most appropriate methodology for gaining an in-depth understanding of perceptions and experiences of dental care access [6, 19]. Furthermore, as we were interested in developing policy and practice recommendations for advanced dental systems, we included only studies conducted in areas of very high HDI. Studies focusing on economic, or undocumented migrants/immigrants, were excluded because these groups have different characteristics or access to healthcare from our population of interest. However, studies that had ASRs as part of a heterogeneous group were included, provided that the results applicable to ASRs were clearly distinguishable. There were no restrictions on year of publication or language.

### Information sources

Five health and social care databases were searched: EMBASE, MEDLINE, CINAHL, SOCINDEX and Dentistry and Oral Sciences Source (DOSS); the searches were conducted on 25th July 2019. Grey literature sources of information (not commercially published) included: Google, EThOS, OpenGrey, the Health Foundation, Social Care Online, United Nations High Commissioner for Refugees (UNHCR), World Health Organization and International Committee of the Red Cross. Thus, we searched resources that were likely to be relevant to our topic, such as charity and organisational websites.

### Search

The searches were developed and conducted by an information specialist (LB). The search strategy that was used in Ovid Embase (and adapted for use in other databases) can be found in Additional file 2. Citation searching of included studies was conducted to supplement the search.

### Study selection

Studies were selected using a two-stage process. Having removed all duplicates via EndNote, two reviewers (MP, RB) independently screened all titles and abstracts against the pre-determined inclusion and exclusion criteria previously described. This process was facilitated using the Rayyan systematic review web application [20]. Following the screening of titles and abstracts, the full texts of potentially relevant articles were retrieved and independently screened by two reviewers (MP, RB). If any discrepancies arose, articles were sent to a third reviewer (AP) until consensus was achieved.

### Data collection process

Data on the included studies were extracted by two independent reviewers (MP, RB) using a pilot-tested form.

### Quality assessment

The Critical Appraisal Skills Programme (CASP) tool [21] was used to critically appraise the studies (MP, RB). Given the lack of consensus on the use of quality appraisal results in qualitative research synthesis [22], no studies were excluded on the basis of quality assessment. Rather, the appraisal was carried out to enhance the transparency of the review. Sensitivity analysis was used to examine whether the inclusion of all studies (irrespective of the quality appraisal results) impacted review findings.

### Data analysis and synthesis

Included studies were initially coded independently by two reviewers (MP, RB) using inductive thematic analysis as outlined by Braun and Clarke [23]. Only findings derived directly from the studies and/or authors’ interpretations (provided these were based on study findings) were coded. The process was facilitated through NVivo software (Version 12).

Identified themes were then deductively organised according to Penchansky and Thomas’s modified access model [14, 24, 25] under the dimensions of affordability, accessibility, accommodation, availability, acceptability (expanded to include the dimension of awareness).

The ENTREQ and PRISMA guidelines were followed for the reporting. Narrative synthesis was used to report the results.

## Results

**Fig. 1:**
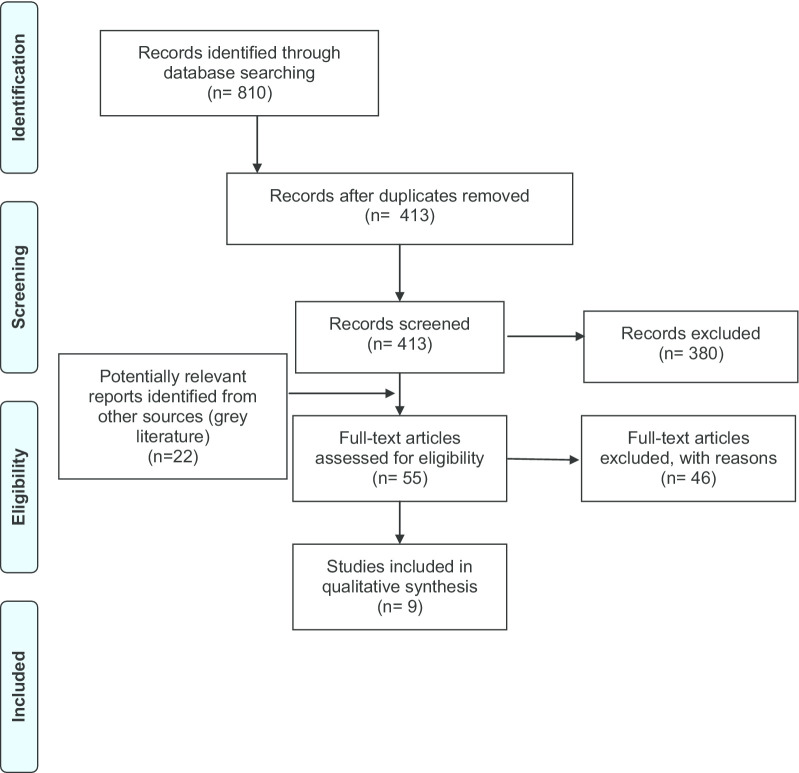
PRISMA flow diagram. *Source*: Moher et al. [44]

The PRISMA Flow diagram shows the search results (Fig. 1).

The initial search of electronic resources identified 810 studies. After deduplications had been removed, 413 papers were screened on title/abstract. Screening of full text papers (N = 33) resulted in the exclusion of 26 papers. In addition to the two reports identified through grey literature sources, 9 papers/reports were included in the review. All studies identified and included in this review were written in English.

The studies were conducted in the US, UK, Canada and Australia. Purposeful sampling was commonly used to recruit participants. Most of the studies (N = 8) used qualitative research methods [25–32], while one adopted a mixed methods approach [33]. Participants were commonly asylum seekers and refugees. The views of other stakeholders, such as nurses, were explored in four studies [27, 29, 32, 33]. When the data was available, the reported number of years residing in the host country by the asylum seekers and/or refugees ranged from 1 month to 13 years. The characteristics of included studies can be seen in Additional file 3.

Critical appraisal of included studies indicated that the aims of the studies were clearly indicated. The methodology and research design were appropriate to the stated objectives. Ethical approval was obtained for most of the studies, but for two [31, 32] this was unclear. The analysis of data was commonly sufficiently vigorous. However, in all but one study the relationship between the researcher and the participants had not been adequately considered. Findings of this review were not influenced by the inclusion of the studies which met less of the appraisal criteria. The results of the critical appraisal can be found in Additional file 4.

The findings on the barriers and enablers to dental care access among ASRs are presented below and in Table 2, under the dimensions of Affordability-Accessibility-Accommodation-Availability-Awareness and Acceptability. Illustrative quotations for each domain are presented in Additional file 5.

**Table 2:** Subthemes identified from data analysis

Dimension	Subthemes
*Affordability*	
Barriers	Cost of dental treatment Lack of finances Ineligibility to healthcare card or insurance
Facilitators	Reduced cost Flexible plan of payment Eligibility to benefits
*Accessibility*	
Barriers	Difficulties travelling to clinics Cultural norms
Facilitators	Mobile services Proximity of clinics Assistance with public transport
*Accommodation*	
Barriers	Limited language skills Lack of insufficient interpretation Long waiting lists and delayed treatments
Facilitators	Interpretation Translated material Collaboration between services
*Availability*	
Barriers	Scarcity of dental services
*Awareness*	
Barriers	Lack of knowledge on service availability, navigation and location Lack of policy awareness and eligbility for care Inconsistent messages between healthcare professionals
Facilitators	Assistance with paperwork Improving awareness of system navigation Discussing oral health and treatment options Training to healthcare professionals and community organisations
*Acceptability*	
Barriers	Dental anxiety Encounters with healthcare teams Oral health beliefs Cultural influences Perceived discrimination
Facilitators	Friendly healthcare team Effective communication and cultural competence

### Affordability

#### Barriers

The high cost of dental treatment, compounded by lack of finances, ineligibility to healthcare cards or insurance, is one of the most common barriers for ASRs to accessing dental care [26, 27, 29, 30]. Furthermore, although in some countries ASRs are entitled to free, or a reduced rate of payment (e.g. in the UK when having the HC2 certificate), some articles reported unexpected patient payment charges [25]. Subsequently, this led to dissatisfaction and affected patients’ trust towards dental and health care services [25].

#### Facilitators

Reducing the cost can enable ASRs to afford dental treatment [26], while streamlining access to free dental care can greatly enhance access and use of services by asylum seekers [32]. As cost is an important influencing factor for this group, a flexible payment plan can help facilitate continuity of care. Research and clinical collaboration can lead to the development of more cost effective dental materials and equipment, and thus reduce the cost of dental care [26]. Changes in healthcare policy harmonising the dental care benefits of ASRs to those offered to social welfare beneficiaries, has also been recommended [26].

### Accessibility

#### Barriers

The geographical location of a dental health service can make travel to a dental appointment challenging. Thus, difficulties in finding dentists that would accept to treat ASRs in geographically accessible areas can pose an obstacle to access [28, 33]. Sometimes, this is compounded by cultural norms (i.e. the need for husband figures to take time off work to drive their wives to the health centre) as well as the resistance of some women to use public transport [33]. Non-mastery of the public transport system and difficulties in adapting to winter conditions were also quoted as reasons for late arrivals or non-attendance to dental appointments [26].

#### Facilitators

Using mobile services and community clinics can be used to enhance dental care access [26]. Considering the location of dental services in regards to public transport can also be of significant benefit when designing healthcare services [26]. Improving awareness of clinic locations and providing assistance with public transport can be a significant support [27].

### Accommodation

#### Barriers

Limited language skills are a common barrier to accessing dental care services. Many participants reported receiving letters in English, some of which may have been appointment reminders, and being unable to read them [29]. Lack of language skills or an interpreter can also make it difficult for patients to explain their dental problems, sometimes leading to perceived inappropriate dental care (i.e. dentist extracting a different tooth to the one causing pain) [25]. The use of interpreters is less common among dentists than compared to GPs, and this is reportedly due to the perceived lack of need by the patients [28]. Long waiting lists and delayed treatment can also lead to disappointment, with people becoming reluctant to proactively seek dental care [29].

#### Facilitators

The use of translators can improve the quality of communication, reduce the risk of misunderstanding and help establish rapport between patients and the dental professional [25, 27]. Translators can also help patients in filling in patient records [26] and provide valid informed consent to the proposed treatment. Providing translated written material has been recommended. Although some may not be able to read in their own language due to literacy skills, others will be able to read the information to them [29]. Having a dentist who speaks the same language is also preferable [26], as it can improve patients’ experiences and confidence in communication without the risk of misunderstanding. Collaboration, effective communication and signposting between services can also improve patients’ access to dental services [29].

### Availability

#### Barriers

The scarcity of public dental services and subsequent difficulties in accessing dental healthcare is a significant barrier faced by ASRs in some host countries. As a result, in combination with lack of awareness and economic difficulties, patients often report attending general physicians for dental issues [25]. Furthermore, heavy workloads among community workers that support asylum seekers and refugees, often leads to de-prioritisation of dental problems; an issue acerbated by scarce resources [27].

#### Facilitators

No facilitators under the availability domain were identified.

### Awareness

#### Barriers

A lack of awareness and understanding about what the dental health care system is, how to access it, where the services are located, or how to find and register with a dentist, can be significant obstacles to accessing dental care [29, 31]. These factors can also result in delays in seeking care. Although asylum seekers and refugees may be given priority to accessing dental care, a lack of awareness of these policies can lead to them not accessing care even with acute dental pain. Similarly, a lack of policy awareness or the availability of services among healthcare professionals caring for this population can also result in false charges and limited signposting and use of services [25, 33]. Inconsistent messages by healthcare professionals (e.g. dentist and doctor advising differently whether it is appropriate to receive dental treatment during pregnancy) can also act as a barrier to seeking care [29].

#### Facilitators

Providing assistance with paperwork and making appointment times clear for ASRs can help facilitate dental access [27]. Housing providers can also help guide new arrivals through the process of registering with a dentist [31]. Discussing patients’ oral health status and treatment options, as well as providing oral hygiene instruction and preventative advice, are highly valued by ASRs [26]. Providing training and resources to community support staff and healthcare professionals, enabling them to identify and signpost pregnant women eligible for priority dental care, and improving their knowledge of oral health and available services can also be beneficial [29]. It is important that services provide consistent messages around oral health [29]. The importance for educating patients on the importance of respecting appointment times has also been emphasised [26].

### Acceptability

#### Barriers

Dental anxiety can lead to delays in seeking care [29]. Miscommunication and differences in expectations between patients and the dental professional can lead to disappointment about the treatment outcome [26] and subsequently affect future use of dental services. The belief that dental care should only be sought in the presence of severe and intolerable pain make this population less likely to seek routine dental care [29]. Cultural attitudes related to the perception of time (e.g. that arriving some minutes later is acceptable for some) can lead to missed, or delayed appointments [26]. In some cultures, male figures are likely to influence women’s health decisions and whether they will access dental treatment [33]. Among pregnant women, there can be fear that dental treatment may lead to negative health consequences for their unborn child [29].

Concerns over differential treatment at policy level regarding dental benefits between ASRs and permanent residents have been reported. Limited dental benefits to this population has been viewed as exclusionary [26].

#### Facilitators

Experiencing a friendly service both by the dental team and the reception staff is important [27]. Having dentists taking into account people’s daily life circumstances and challenges, and treating them with compassion, kindness and dignity are highly valued by patients [26].

## Discussion

### Statement of principal findings

This is the first review to systematically synthesise the barriers and facilitators to dental care access among ASRs in countries of very high development. Our review findings suggest that ASRs encounter significant challenges to accessing dental care in their host countries, with dominant themes including affordability, limited language skills, insufficient interpretation, limited knowledge of the healthcare system and healthcare rights related to the immigration status, and miscommunication within and between healthcare teams. The findings of this review can enable policy makers and healthcare professionals to develop appropriate policies and interventions to address the above challenges and improve dental care access for ASRs. Furthermore, the facilitators identified could inform the measures taken, to ensure that they meet the needs of this particular group.

In the present review, access to dental care among ASRs was viewed via the lens of Pechansky and Thomas’s [14] modified model, where access is influenced by both the characteristics of the population and the healthcare system. Themes within the domains of affordability, accommodation and awareness were identified as significant barriers to accessing care. Evidently, the themes identified can act independently but can also interact to influence access. For example, limited language skills can affect communication between the patient and the dental team and subsequently lead to disappointment over the outcome of treatment and reduce uptake of care.

### Comparison with existing literature

A scoping review into the oral health of ASRs identified the healthcare policy of the host country as a key factor influencing access to dental care among this group [7], echoing the findings of our review. However, access to care was only one of the objectives of that review and acted as a theme. The present systematic review provides a more in-depth analysis in relation to the barriers and facilitators of dental care access among this population. These appear to be similar to those of other vulnerable populations in the host country, for example those experiencing homelessness [34]. However, while accessibility is a common barrier to care, some differences exist with regard to how the different population characteristics may affect access. For example, for people experiencing homelessness, the complexity associated with the lived experience of homelessness appears to be a significant factor influencing uptake of care, while in the case of ASRs, language skills are more pertinent. This demonstrates the need to investigate each group’s needs independently before collating the findings between different population groups. Furthermore, some of the findings of the review attributable to language difficulties also apply to patients fluent in English due to the challenges of diagnosis, rather than communication difficulties. An example would be a patient having a tooth extirpated (nerve/pulp removed) rather than restored (caries/decay removed only) due to a patient not being able to report on the characteristics of the pain (especially regarding duration of pain in response to stimuli).

The findings of the present review also indicate that the barriers and facilitators to accessing dental care for ASRs are conceptually similar to those identified for other healthcare settings (e.g. primary healthcare, mental health care) [17]. Thus, the findings can also be used to complement other healthcare policies for this population group. In parallel, findings of reviews in other settings/systems can be examined for applicability in the dental healthcare system and could be used to inform service design and delivery for this population.

### Implications for research, practice, education and policy

Undoubtedly, changes to dental health systems and policies within the host countries are required if the needs of this population are to be met. For example, in the UK context, when it comes to the use of interpreters there is an important element of cost involved which may affect their use. In National Health Service (NHS) practices where dentists are remunerated based on the volume and complexity of the work that they undertake, spending time using an interpreter may drive up the costs of providing treatment for these patients. However, even when existing policies facilitate access to dental care, they do not always translate to improvement of access [6, 35]. It has been shown that even when provided free of charge, utilisation of services can remain low [35]. Therefore, it is clear that when developing programmes to widen access to this population, more than one barriers will need to be considered at any given time. Furthermore, improving awareness of rights and eligibility through community gatekeepers (in the case of ASRs) and training (for healthcare professionals) is paramount.

Cultural attitudes to dental care among ASRs that are in general not aligned with western patient centred/shared decision making approaches (where there is a strong emphasis on prevention), highlights the need to raise awareness of oral health in this population. This is further supported by studies which show that culturally based oral health beliefs and practices, and knowledge gaps, influence this population’s oral health status and attitudes towards dental care, respectively [7]. For example, in a study conducted in Canada [36], parents/caregivers of refugee children believed that children should go to the dentist only when experiencing pain. Education regarding the fact that dental diseases are largely preventable and that the role of the dentist should not be paternalistic, is therefore important. Incorporating ‘cultural competence’ defined as ‘*the ability of systems to provide care to patients with diverse values, beliefs and behaviours, including tailoring delivery to meet patients’ social, cultural and linguistic needs’* into policy and practice can optimise quality of care towards asylum seekers and refugees [6] and ensure that care becomes more equitable [37]. Elements incorporated in cultural competence, such as effective communication and understanding of patients’ backgrounds and needs, have been shown to improve dental experiences, alleviate fears and improve confidence in the treatment procedure and outcome [26]. This had the potential to improve compliance and treatment outcomes. In contrast, perception of unfair treatment by the dentists, can lead to avoidance of the use of services [26].

Therefore, developing a funding model where a compassionate workforce can take the patients’ social contexts into account,is paramount to encouraging continuous use of services and achieving patient-centred care. Healthcare professionals need to be adequately resourced and trained in the provision of care to this population, both in terms of ASRs’ rights to accessing care but also with regard to tailoring treatment to the specific needs of this particular population.

Enabling undergraduate dental students to conduct community engagement activities with vulnerable groups, is an ideal time and pathway to start instilling empathy and understanding of the factors that may influence the ability of vulnerable groups to care for themselves and seek care [38, 39].

### Strengths and weaknesses of the study

A systematic and comprehensive approach of collecting and identifying the papers was used, and therefore it is unlikely that any studies were missed. The analytical framework used to synthesise the findings enabled us to provide a rigorous analysis of the barriers and enablers to dental care for ASRs. A strength of synthesis of qualitative data, is that themes may be more transferable to other contexts and have greater potential to inform policy and practice, in comparison to individual qualitative studies [6, 40]. Our findings may also be transferable to other healthcare settings, as indicated by other systematic reviews which explored experiences of this population in the host countries [4, 17]. In addition, the methodology used provides a transparent link between the primary studies and the conclusions drawn in this review. By involving more than one reviewer in all study stages selection bias was reduced.

However, limitations of this review must also be acknowledged. As the characteristics of healthcare systems and dental services for this population vary between countries, some findings may not be transferable to all countries of very high development. By providing as many details as possible about the context of our work and characteristics of the studies included, others can assess whether the findings of this review could transferred to other contexts and populations. In addition, it was common for themes to fit to more than one domain of access, consistent with other studies that employed a similar methodological approach [25]. As our primary aim was to identify the barriers and facilitators to dental care access as perceived/experienced by ASRs and those who support them, only qualitative studies were sought. Although, it is often proposed that qualitative research is not generalisable [40], a strong case has been made for the potential of qualitative studies to inform policy and practice. On the other hand, quantitative studies have their own limitations. They use highly structured methods and participants need to choose from fixed responses. We recognise that adopting a mixed methods approach (i.e. including both quantitative and qualitative studies), could have maximised the potential of our findings to inform policy and practice [41]. Lastly, although there is a possibility that studies that used terms such as ‘migrants’ instead of ‘ASRs’ may have been missed, the risk was minimised by also conducting a citation searching.

### Unanswered questions and future research

The review found that research exploring the views and experiences of dental teams providing care to ASRs is very limited. Furthermore, more in-depth studies exploring the mechanism by which the experiences of ASRs in receiving care and the refugee journey can influence access to dental care is warranted. A better understanding of the barriers and facilitators to dental care access and provision of care to this population is paramount to improving quality of care and providing healthcare teams with the appropriate resources to achieve this.

Although there are reports that previous traumatic experiences may render seeking and receiving dental care for this population particularly distressing [42, 43], evidence on how previous trauma may affect use and utilisation of dental care by this population is lacking. The lack of evidence may well be due to ethical dilemmas with investigating the impact of trauma. However, exploring means to identify the impact of torture and persecution on seeking and utilizing dental care, can allow for pathways towards improving trauma-informed care to this population.

Investigating the barriers and facilitators to dental care access among ASRS in countries of lower HDI, could complement the findings of the current review.

## Conclusions

Both population and healthcare characteristics influence access to dental care for ASRs in host countries of very high development. Affordability, awareness and accommodation are the main domains influencing utilisation of care. The diverse needs of this population need to be recognised by policy makers, commissioners and practitioners alike and appropriate measures to improve access to care need to be developed.

There is a need to develop and evaluate models for engagement of this population with dental services. Further studies exploring the views of ASRs and healthcare professionals are warranted. The elements identified in this review can help improve practice when engaging with this group, but can also assist policy and commissioning development towards improving healthcare provision and dental care access for ASRs.

## Supplementary information

**Additional file 1.** Countries of very high HDI. List of eligible countries.

**Additional file 2.** Search strategy that was used in Ovid Embase (and adapted for use in other databases). The search strategy used in Ovid EMBASE.

**Additional file 3.** Table of characteristics. Characteristics of studies included in the systematic review.

**Additional file 4.** Critical appraisal results. Results of the critical appraisal of the included studies.

**Additional file 5.** Illustrative quotations for each domain. Illustrative quotations for each of the six domains.

## Data Availability

All data generated or analysed during this study are included in this published article [and its supplementary information files].

## Abbreviation

ASRs: Asylum seekers and refugees
