# Supplementary material for: Barriers and facilitators to dental care access among asylum seekers and refugees in highly developed countries: a systematic review
Source: BMC Oral Health. 2020 Nov 25;20:337. doi: 10.1186/s12903-020-01321-1 (PMC7687682; doi:10.1186/s12903-020-01321-1)
Supplement: Supplementary file 2 — Additional file 2. Search strategy that was used in Ovid Embase (and adapted for use in other databases). The search strategy used in Ovid EMBASE. [file 12903_2020_1321_MOESM2_ESM.docx]

**Additional File 2:** Search strategy that was used in Ovid Embase (and adapted for use in other databases)

# Searches Results

1 refugee/ 11424

2 asylum seeker/ 763

3 "refugee*".ab, kw, ti. 10718

4 (asylum adj3 seek*).ab, kw, ti. 2013

5 (forced adj3 migrat*).ab, kw, ti. 332

6 (displaced adj3 (person* or people or population*)).ab, kw, ti. 1285

7 humanitarian protection.ab, kw, ti. 8

8 1 or 2 or 3 or 4 or 5 or 6 or 7 15626

9 dental health/ 3506

10 dental procedure/ 21102

11 exp tooth disease/ 202963

12 exp dentist/ 23049

13 (oral adj3 (health or hygiene or care)).ab, kw, ti. 38464

14 dental.ab, kw, ti. 205008

15 (tooth adj3 (health or hygiene or care)).ab, kw, ti. 539

16 (teeth adj3 (health or hygiene or care)).ab, kw, ti. 741

17 dentist*.ab, kw, ti. 66359

18 or/9-17 394863

19 8 and 18 286
