# Supplementary material for: Barriers and facilitators to dental care access among asylum seekers and refugees in highly developed countries: a systematic review
Source: BMC Oral Health. 2020 Nov 25;20:337. doi: 10.1186/s12903-020-01321-1 (PMC7687682; doi:10.1186/s12903-020-01321-1)
Supplement: Supplementary file 5 — Additional file 5. Illustrative quotations for each domain. Illustrative quotations for each of the six domains. [file 12903_2020_1321_MOESM5_ESM.docx]

**Additional File 5.** Illustrative quotations for each domain

| **Domain** |  | **Quotation and reference** |
| --- | --- | --- |
| **Affordability** | Barrier | ‘When I went to my dentist, they ask me give £100. They charge me, and that was really a shock, even besides having this, you know, HC2 form. To be honest, very scary when they ask so much money. That was a really bad experience.’ (participant ASR)^1^  ‘Because it’s expensive we’re not going but some of the others, they are going for treatment overseas like in Afghanistan, Pakistan it is cheap.’ (participant ASR)^2^ |
|  | Enabler | Participants expressed the need for a reduction in the cost of dental treatment. This would make it affordable for people like themselves who cannot afford the current fees. (authors)^3^  For asylum seekers, streamlining access to free care and finding innovative programs to subsidizing secondary care would greatly improve access to and use of the already existing services. (author)^4^ |
| **Accessibility** | Barrier | the non-mastery of the public transport system in Montreal and difficulties in adapting to winter conditions were additional reasons why new humanitarian immigrants arrived late or missed their dental appointments.(authors)^3^ |
|  | Enabler | Three participants from Syria, Iraq, and Mexico suggested the use of specially equipped vans to provide basic dental care in disadvantaged neighbourhoods. In addition, they felt the government should open dental clinics that provide services at reduced rates to humanitarian migrants and other poor populations. (authors)^3^ |
| **Accommodation** | Barrier | Some participants reported the HC2 application as a lengthy process. (authors)^1^ |
|  | Enabler | Translated written information was also suggested although it was recognised that not everyone could read in their own language but most knew someone who could translate for them. (authors)^2^ |
| **Availability** | Barrier | There was general agreement that access to dental care was difficult, and many had not ever tried to get a dentist. (authors)^5^  Many of the participants related attending the GP with dental problems. This phenomenon is widespread, due to scarcity of NHS dentist (authors)^1^ |
|  | Enabler | None identified |
| **Awareness** | Barrier | A lack of awareness of the structure and function of NHS services emerged as a dominant theme.(author)^1^  Midwives were shocked to realise that they were not aware that a dental service was available within the same organisation in which they were employed. (authors)^2^ |
|  | Enabler | Participants expressed satisfaction … when they had assistance with paperwork..  (authors)^6^ |
| **Acceptability** | Barrier | Four participants narrated incidents where they felt misunderstood, humiliated, or not provided with appropriate care. Misinterpretation or misunderstanding of the IFHP policy was at the core of these unfortunate situations, (authors)^3^ |
|  | Enabler | ‘I had two kids when I arrived, two and a half and three years, they had really bad teeth, so I had to go to hospital, they had X-ray, and they had to take out all the teeth. And they had two crowns in, now eight and nine years, and I now take them regularly for check up. It was a nice experience; they were kind to me.’ (participant ASR)^6^ |

**References**

1. Kang C, Tomkow L, Farrington R. Access to primary health care for asylum seekers and refugees: a qualitative study of service user experiences in the UK. The British Journal of General Practice. 2019;69(685):e537–e545.
2. Riggs E, Yelland J, Shankumar R, Kilpatrick N. 'We are all scared for the baby': Promoting access to dental services for refugee background women during pregnancy. BMC Pregnancy and Childbirth. 2016;16(1):12.
3. Keboa MT, Hovey R, Nicolau B, Esfandiari S, Carnevale F, Macdonald ME. Oral healthcare experiences of humanitarian migrants in Montreal, Canada. Canadian Journal of Public Health.2019;110(4):453-461.
4. Ineza D. Barriers to Healthcare Access for New Mainers. Brunswick: Bowdoin College; 2019.
5. Campion P, Brown SR, Thornton-Jones H. After Wilberforce : an independent enquiry into the health and social needs of asylum seekers and refugees in Hull. Hull: NHS Hull; 2010.
6. Nicol P, Al-Hanbali A, King N, Slack-Smith L, Cherian S. Informing a culturally appropriate approach to oral health and dental care for pre-school refugee children: a community participatory study. BMC Oral Health. 2014;14:69.
